# Obesity Increases In-Hospital Mortality of Acute Type A Aortic Dissection Patients Undergoing Open Surgical Repair: A Retrospective Study in the Chinese Population

**DOI:** 10.3389/fcvm.2022.899050

**Published:** 2022-07-12

**Authors:** Xiaogao Pan, Zhenhua Xing, Guifang Yang, Ning Ding, Yang Zhou, Xiangping Chai

**Affiliations:** ^1^ Department of Emergency Medicine, Second Xiangya Hospital, Central South University, Changsha, China; ^2^ Emergency Medicine and Difficult Diseases Institute, Second Xiangya Hospital, Central South University, Changsha, China; ^3^ Emergency Department, Changsha Central Hospital, University of South China, Changsha, China

**Keywords:** obesity, body mass index, aortic dissection, open surgical repair, in-hospital mortality

## Abstract

**Objective:**

The prevalence of obesity is increasing worldwide, and the role of the obesity paradox in cardiovascular surgery remains controversial. In this study, we redefined obesity according to the Chinese criteria and examined the relationship between obesity and in-hospital mortality in patients with acute type A aortic dissection (AAD) undergoing open surgical repair.

**Materials and Methods:**

A total of 289 patients with AAD (between 2014 and 2016) were divided into the non-obese group and obese group for correlation analysis, general information, demographic factors, blood biochemistry, surgical details, and complications, which were used as covariates. Survival was estimated by the Kaplan–Meier method, and any differences in survival were evaluated with a stratified log-rank test. Least Absolute Shrinkage and Selection Operator (LASSO) regression and logistic regression were used to evaluate the effect and interaction of obesity on surgical mortality.

**Results:**

All the 289 patients had a mean age of 48.64 (IQR 44.00–55.00) and 74.39% were men. Of the 289 patients, 228 were non-obese (78.89%) and 61 were obese (21.11%). Patients with obesity were younger and more prone to unstable blood pressure [systolic blood pressure (SBP) and diastolic blood pressure (DBP)], preoperative hypoxemia and delirium, prolonged operative time, and surgical wound deep infection (*p* < 0.05). In the fully adjusted model, we observed an increased risk of in-hospital mortality in patients with obesity after fine-tuning other covariates including age and sex (HR = 2.65; 95% CI = 1.03 to 6.80; *p* = 0.042). The interaction suggested that obesity was more likely to cause death in elderly patients (age ≥ 60), although it was more common in younger patients (test for interaction, *p* = 0.012).

**Conclusion:**

Obesity, interacting with age, increases the risk of in-hospital mortality in patients with AAD undergoing open surgical repair. Although more verification is needed, we believe these findings provide further evidence for the treatment of AAD.

## Introduction

Over the past three decades, China has experienced rapid economic development and nutrition transition, and the prevalence of overweight and obesity in China has increased 2 to 3 times since the 1980s (1). The pandemic of obesity is rising worldwide, affecting individuals of all ages, involving various diseases, and increasing the economic burden. More than two-thirds of deaths related to high body mass index (BMI) were due to cardiovascular disease (2). However, the existence of a protective obesity paradox makes the role of obesity in cardiovascular surgeries uncertain (3), as is the case in open surgical repair of acute Stanford type A aortic dissection (AAD).

In recent years, increasing efforts have been made to assess the trends and effects of BMI within and across nations (4). Other studies have attempted to compare the potential effects of high BMI on a variety of aortic surgery outcomes. These efforts, while useful, appeared to deviate from daily clinical practice. In these studies, patients with obesity appeared to have a higher risk of acute kidney injury (AKI) (5), hypoxemia (6), acute lung injury (7), and prolonged intubation (8, 9) in the perioperative period of AAD, who also have a higher prevalence of several risk factors for AAD according to previous researches (10, 11), such as hypertension, hyperlipidemia, and stroke. However, what is puzzling is that the results of these studies all showed that obesity was not related to AAD mortality. Are we omitting the role of the perioperative “obesity paradox”? Or did the obesity interaction mask a single effect?

On the one hand, most of the patients in the study Kreibich et al. (9) underwent hemiarch replacement surgery (9), which was different from the current mainstream total arch replacement (12, 13). On the other hand, the unified international BMI classification is not applicable to the Chinese population due to differences in ethnicity and living habits, and the overall proportion of BMI ≥ 30 kg/m^2^ may be about 3% in China (14, 15), while it may exceed 10% in other regions (2). Based on these findings, World Health Organization (WHO), the International Association for the Study of Obesity, and the International Obesity Task Force have suggested lower BMI cutoffs for overweight and obesity in Asian populations (16). Thus, the use of the international BMI classification for comparisons might confound any association between body weight and mortality of AAD. The influence of obesity on perioperative or open surgical repair AAD remains unclear in the Chinese population based on Chinese standards. This study aims to evaluate the effect and interaction of obesity with gender, age, and blood pressure on in-hospital mortality.

## Materials and Methods

### Patients

This was a retrospective, observational research consisting of 289 in-patients operated at the hospital from January 2014 to December 2016. All patients presented with AAD and were treated by open surgical repair. We non-selectively and consecutively collected data for all participants at the Second Xiangya Hospital of Central South University, Changsha, Hunan, China. Anonymous data were compiled from the electronic hospital medical record system. Ethical approval for the study was provided by the hospital’s institutional review board. Informed consent was waived because the study was retrospective.

### Inclusion/Exclusion

**FIGURE 1:**
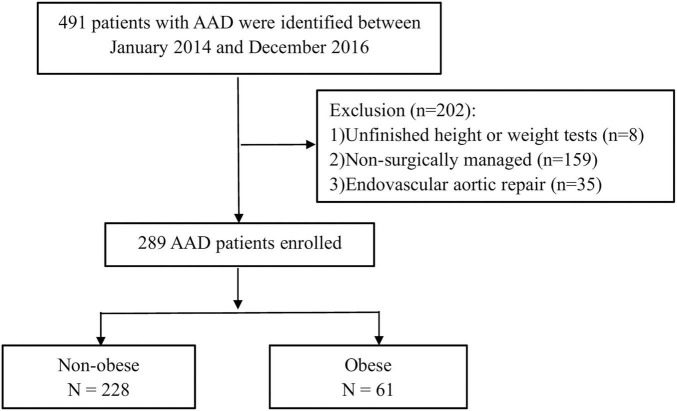
A flowchart of patient enrollment.

The inclusion criteria were hospital admission for patients with acute type AAD within ≤14 days after symptoms onset. The following were used as exclusion criteria: (1) unfinished height or weight test; (2) non-surgically managed condition; and (3) endovascular aortic repair (Figure 1).

### Standard Measures

The diagnosis was mainly based on 2014 ESC guidelines on the treatment and diagnosis of aortic ailments (17). Any dissection that involved the ascending aorta with presentation within 14 days of symptom onset was defined as AAD. The diagnosis of AAD was confirmed by imaging like computed tomography (CT) or magnetic resonance imaging (MRI). Admission BMI (kg/m^2^) measured at baseline was calculated as weight in kilograms divided by the square of height in meters (1). For correlation analysis between obesity and mortality, patients were divided into two groups according to the Chinese criterion (WS/T 428–2013) (10): non-obese (BMI: < 28 kg/m^2^) and obese (BMI: ≥ 28 kg/m^2^). Overall survival was defined as the time from surgery until death from any cause. Systolic blood pressure (SBP), diastolic blood pressure (DBP), and heart rate were measured half an hour after pharmacotherapy. Pre-operative and post-operative complications were diagnosed by clinical examination and confirmed by CT angiography. Other covariates involving general information, demographic data, blood biochemistry, medical imaging examination, and treatment variables that can affect in-hospital mortality were confirmed based on clinical characteristics.

### Surgical Procedure

The detailed surgical procedures have been previously described in the literature (18, 19). To summarize in brief, cardiopulmonary bypass (CPB) was instituted through the right atrial graft to the right axillary artery by cannulation. Femoral artery cannulation was used if the dissection involved the right axillary artery or if the pumping pressure was too high. The aortic repair included replacing the entire ascending aorta, when the aortic root or valve was also involved, while the Bentall procedure, the Wheat procedure, or the Valsalva sinuplasty, among others, was performed simultaneously. Coronary artery bypass grafting was performed in patients with dissection involving the coronary artery or in patients with pre-operative severe coronary heart disease (20).

### Missing Data Addressing

We performed multiple multivariable imputations to address missing data in order to maximize statistical power and minimize bias. Five imputed datasets with chained equations were created using R-package mice (21). Our multiple imputations of the dataset were mainly based on the following principles: (1) there were no missing data for the primary outcome; (2) replacement of categorical variables was not advisable, as the plausibility is still debated; and (3) 5% missingness is suggested as a maximum upper threshold below which multiple imputations provides benefit. Sensitivity analysis found no significant differences between the generated complete data and raw data. Thus, all multivariable analysis results based on the imputed datasets were combined with Rubin’s rules (22, 23).

### Statistical Analysis

Continuous variables were expressed as median (interquartile range, IQR). A *t*-test and the Mann–Whitney *U*-test were used for parametric and non-parametric data, respectively. Categorical variables were expressed as frequencies and compared using Fisher’s precision probability test or Chi-square analysis. Survival was estimated by the Kaplan–Meier method, and any differences in survival were evaluated with a stratified log-rank test. Least Absolute Shrinkage and Selection Operator (LASSO) regression was applied to minimize the potential collinearity of variables measured from the same patient and over-fitting of variables (24). Multivariable analyses with the Cox proportional hazards model were used to estimate the simultaneous effects of prognostic factors on survival. Interactions with prognostic factors were also examined with the Cox proportional-hazards model. EmpowerStats (X&Y Inc Solutions, Boston, MA, United States)^1^ and R version 4.0.5^2^ were used for statistical analyses. A *p*-value ≤ 0.05 was considered statistically significant.

## Results

### Characteristics of Baseline

The 289 patients had a mean age of 48.64 and 74.39% of them were men. Of the 289 patients, 228 were non-obese (78.89%) and 61 were obese (21.11%). The differences in baseline characteristics are presented in Table 1. Compared with non-obese patients, patients with obesity on admission appeared to be younger, had lower DBP, had a higher probability of hypertension, and were more difficult to obtain expected blood pressure (100∼120 mmHg) from half an hour after pharmacotherapy. A total of eight patients with Marfan syndrome were all non-obese, but the difference between the two groups was not statistically significant (Fisher’s precision probability test, *p* = 0.138). There was also no significant difference between the two groups in terms of gender, diabetes mellitus, stroke, and bicuspid aortic valve (*p* > 0.05).

**TABLE 1:** Baseline characteristics of the patients.

Characteristic	Overall (*n* = 289)	Non-obese (*n* = 228)	Obese (*n* = 61)	*p*-Value
Age, year	48.64 (44.00–55.00)	49.30 (44.75–55.00)	46.18 (39.00–53.00)	0.015
Age ≥ 60.00	38 (13.15)	31 (13.60)	7 (11.48)	0.663
**Gender**				0.838
Men	215 (74.39)	169 (74.12)	46 (75.41)	
Women	74 (25.61)	59 (25.88)	15 (24.59)	
SBP, mmHg	133.27 (114.00–154.00)	131.75 (114.00–148.25)	138.97 (115.00–162.00)	0.074
<100.00	30 (10.38)	20 (8.77)	10 (16.39)	0.083
100.00–120.00	74 (25.61)	66 (28.95)	8 (13.11)	0.012
>120.00	185 (64.01)	142 (62.28)	43 (70.49)	0.235
DBP, mmHg	75.73 (66.00–84.00)	76.74 (69.75–86.00)	71.98 (57.00–80.00)	0.034
<60.00	40 (13.84)	23 (10.09)	17 (27.87)	<0.001
60.00–90.00	195 (67.47)	158 (69.30)	37 (60.66)	0.201
>90.00	54 (18.69)	47 (20.61)	7 (11.48)	0.104
Heart rate/min	80.83 (70.00–90.00)	80.81 (71.00–90.00)	80.90 (65.00–91.00)	0.849
<60.00	21 (7.27)	20 (8.77)	1 (1.64)	0.057
**History of**				
Hypertension	133 (46.02)	115 (50.44)	41 (67.21)	0.020
Diabetes mellitus	156 (53.98)	8 (3.51)	4 (6.56)	0.289
Marfan	8 (2.77)	8 (3.51)	0 (0.00)	0.138
Stroke	20 (6.92)	14 (6.14)	6 (9.84)	0.312
Bicuspid aortic valve	6 (2.08)	5 (2.19)	1 (1.64)	0.788

*Data are presented as n (%) or mean (IQR).*

*SBP, systolic blood pressure; DBP, diastolic blood pressure.*

### Clinical Pre-operative Data

Obese patients have higher red cell volume distribution width (RDW) and neutrocyte lymphocyte ratio (NLR). Two patients with left ventricular ejection fraction (LEVF) <20% were obese, and the difference was statistically significant (Fisher’s precision probability test, *p* = 0.044). The obese group also appeared to be more susceptible to hypoxemia (6.58 vs. 16.39%, *p* = 0.015) and delirium (1.75 vs. 6.56%, *p* = 0.043). Patients presented with similar rates of pre-operative C-reactive protein (CRP), creatinine, platelet lymphocyte ratio (PLR), aortic regurgitation, LEVF, pericardial effusion, ascending aortic diameter (*p* > 0.05) (Table 2).

**TABLE 2:** Clinical pre-operative data.

Variable	Overall (*n* = 289)	Non-obese (*n* = 228)	Obese (*n* = 61)	*P*-Value
**Pre-operative blood test**				
CRP, mg/L	59.12 (17.30–92.10)	57.39 (17.30–86.20)	65.31 (15.93–106.33)	0.393
Creatinine, umol/L	100.51 (71.30–110.10)	98.54 (70.60–108.68)	107.61 (83.80–112.30)	0.370
RDW	14.02 (13.67–16.00)	13.44 (12.70–14.00)	14.80 (13.40–15.40)	0.019
PLR	167.15 (89.92–210.31)	169.63 (89.70–216.16)	157.25 (95.24–207.41)	0.375
NLR	11.10 (6.52–14.90)	10.83 (6.34–14.13)	12.19 (7.41–17.99)	0.022
**Transthoracic echocardiography**				
Aortic regurgitation				0.812
None	106 (36.68)	82 (35.96)	24 (39.34)	
Mild	145 (50.17)	116 (50.88)	29 (47.54)	
Medium	29 (10.03)	22 (9.65)	7 (11.48)	
Severe	9 (3.11)	8 (3.51)	1 (1.64)	
Left ventricular ejection fraction	66.36 (62.00–70.00)	66.66 (63.00–70.25)	65.21 (62.00–69.00)	0.110
20.00–40.00	2 (0.69)	0 (0.00)	2 (3.28)	0.044
40.00–60.00	38 (13.15)	28 (12.28)	10 (16.39)	0.399
>60.00	251 (86.85)	200 (87.72)	51 (83.61)	0.399
Pericardial effusion	77 (26.64)	58 (25.44)	19 (31.15)	0.370
Ascending aortic diameter, mm	46.13 (41.00–50.00)	46.09 (40.00–50.00)	46.28 (41.00–49.00)	0.868
**Pre-operative complications**				
Hypoxemia	25 (8.65)	15 (6.58)	10 (16.39)	0.015
Delirium	8 (2.77)	4 (1.75)	4 (6.56)	0.043

*Data are presented as n (%) or mean (IQR).*

*CRP, C-reactive protein; RDW, red cell volume distribution width; PLR, platelet lymphocyte ratio; NLR, neutrocyte lymphocyte ratio.*

### Intraoperative Details

Operative details are shown in Table 3. Overall, Bentall procedure (88.23%), total arch replacement (93.43%), and elephant trunk procedure (93.77%) were the main surgical procedures. There were seven (2.42%) patients who underwent aortic valve replacement and thirty (10.38%) patients who underwent coronary artery bypass graft (CABG). Patients with obesity required a longer duration of surgical procedure, CPB, and hypothermic circulatory arrest. No statistical difference was found between the different groups in root and aortic arch procedure, elephant trunk procedure, concomitant procedure, time of aortic cross-clamp, and ventricular fibrillation (*p* > 0.05).

**TABLE 3:** Intraoperative details.

Variable	Overall (*n* = 289)	Non-obese (*n* = 228)	Obese (*n* = 61)	*p*-Value
**Root procedure**				
Bentall	255 (88.23)	206 (90.00)	49 (80.00)	0.063
David	26 (9.00)	21 (9.21)	5 (8.20)	0.102
Wheat	8 (2.77)	6 (2.63)	2 (3.29)	0.086
**Aortic arch procedure**				
Total arch replacement	270 (93.43)	210 (92.11%)	60 (98.36)	0.080
Hemiarch replacement	9 (3.11)	8 (3.51%)	1 (1.64)	0.690
Elephant trunk procedure	271 (93.77)	211 (92.54)	60 (98.36)	0.095
**Concomitant procedure**				
Aortic valve replacement	7 (2.42)	5 (2.19)	2 (3.28)	0.624
CABG	30 (10.38)	22 (9.65)	8 (13.11)	0.431
**Time of operation**				
Total operation, min	538 (450–650)	528 (430–645)	574 (485–660)	0.012
Aortic cross-clamp, min	124 (84–153)	122 (83–148)	128 (101–159)	0.376
Cardiopulmonary bypass, min	262 (197–318)	255 (192–311)	289 (255–329)	0.004
Hypothermic circulatory arrest, min	32 (0–54)	30 (0–52)	39 (0–60)	0.028
Ventricular fibrillation, seconds	36 (15–54)	37 (17–55)	33 (0–54)	0.255

*Data are presented as n (%) or mean (IQR).*

*CABG, coronary artery bypass graft.*

### Post-operative Characteristics

The outcome characteristics are summarized in Table 4. There was no statistical difference between the two groups in RDW, PLR, NLR, respiratory infection, renal replacement therapy, paraplegia, temporary neurological dysfunction, stroke, and hospital stay. However, it seemed that patients with obesity were more likely to develop surgical wound deep infection (2.22 vs. 8.62%, *p* = 0.019), higher intensive care unit (ICU) stay rate (0.37 vs. 0.59, *p* < 0.001), and in-hospital mortality (11.4 vs. 21.31%, *p* = 0.043, Supplementary Figure 1).

**TABLE 4:** Post-operative Characteristics.

Variables	Overall (*n* = 289)	Non-obese (*n* = 228)	Obese (*n* = 61)	*p*-Value
**Post-operative blood test**				
RDW	14.97 (13.67–16.00)	15.06 (13.70–16.00)	14.63 (13.50–15.60)	0.172
PLR	153.63 (86.44–201.61)	156.52 (91.27–213.25)	141.26 (85.71–189.90)	0.244
NLR	11.78 (8.84–14.15)	11.75 (8.93–14.30)	11.89 (8.71–14.03)	0.844
**Complications**				
Respiratory infection	36 (12.77)	29 (12.89)	7 (12.28)	0.902
Surgical wound deep infection	10 (3.53)	5 (2.22)	5 (8.62)	0.019
Renal replacement therapy	53 (18.79)	40 (17.78)	13 (22.81)	0.385
Paraplegia	8 (2.82)	6 (2.64)	2 (3.51)	0.663
Temporary neurological dysfunction	12 (4.26)	8 (3.56)	4 (7.02)	0.247
Stroke	13 (4.58)	10 (4.41)	3 (5.26)	0.782
Hospital stay, day	19.96 (14.00–23.00)	20.50 (15.00–23.00)	17.95 (12.00–21.00)	0.066
ICU stay rate	0.42 (0.27–0.53)	0.37 (0.25–0.47)	0.59 (0.50–0.68)	<0.001
In-hospital mortality	39 (13.49)	26 (11.40)	13 (21.31)	0.043

*Data are presented as n (%) or mean (IQR).*

*RDW, red cell volume distribution width; PLR, platelet lymphocyte ratio; NLR, neutrocyte lymphocyte ratio; ICU, intensive care unit.*

### Kaplan–Meier Analysis in Different Groups

**FIGURE 2:**
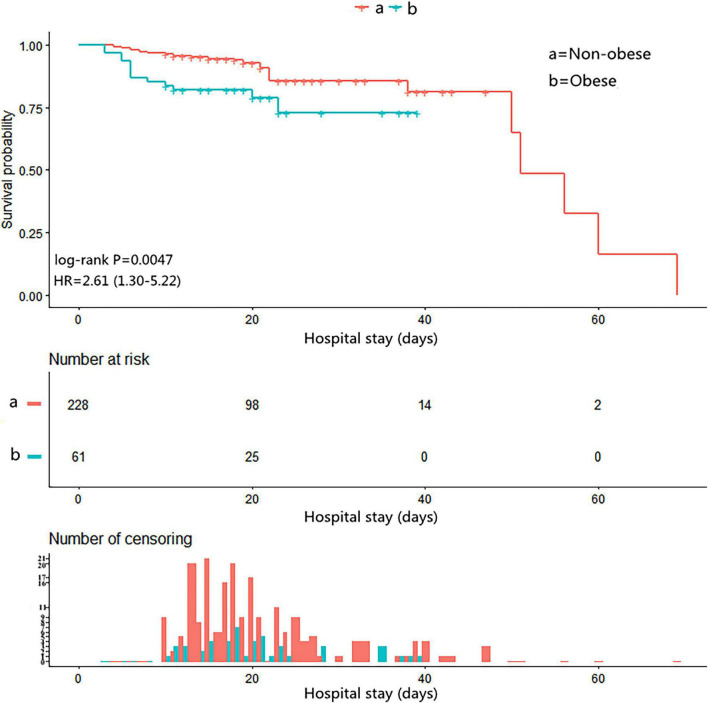
The Kaplan–Meier curves for in-hospital survival in the chronological trend after open surgical repair in AAD.

Follow-up data were available for 289 patients. The Kaplan–Meier analysis showed that the cumulative survival rate of patients with obesity in the hospital was significantly reduced (log-rank test *p* = 0.0047, Figure 2).

### Adjusted and Unadjusted Models for Obesity and In-Hospital Mortality

A total of 59 variables measured at the hospital (Tables 1–4) were included in the LASSO regression. After LASSO regression selection (Supplementary Figure 2), 13 variables remained significant predictors of in-hospital mortality, including clinical features and test results: obesity, gender, age ≥ 60, SBP, DBP, heart rate, pre-operative delirium, LEVF (20–40,%), time of ventricular fibrillation during surgery, post-operative NLR, post-operative PLR, renal replacement therapy, and stroke.

**FIGURE 3:**
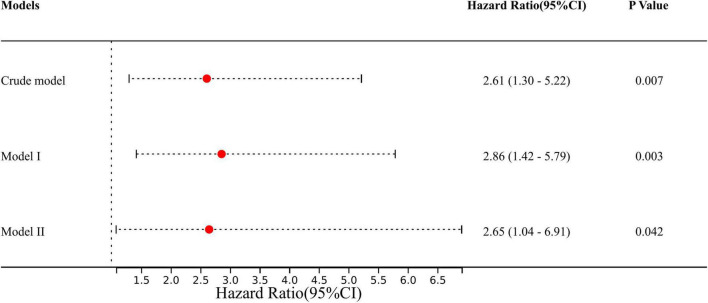
The effect of obesity on outcomes following multivariable analysis based on the Cox proportional-hazard model. Crude model: adjusted for none. Model I: adjusted for: gender and age. Model II: adjusted for gender, age, SBP, DBP, heart rate, pre-operative delirium, LEVF 20–40%, time of ventricular fibrillation during surgery, aortic valve replacement, CABG, post-operative PLR, post-operative NLR, renal replacement therapy, and stroke. SBP, systolic blood pressure; DBP, diastolic blood pressure; LEVF, left ventricular ejection fraction; CABG, coronary artery bypass graft; PLR, platelet lymphocyte ratio; NLR, neutrocyte lymphocyte ratio.

We defined the above 12 variables other than obesity as covariates affecting in-hospital mortality in patients with AAD. We constructed three models to analyze the independent effects of obesity on in-hospital mortality (univariate and multivariate) based on the proportional hazards model. The hazard ratio (HR) and 95% confidence intervals (CI) were listed in Figure 3. In the full model (model II), after adjusting for all covariates, patients with obesity had a higher risk of in-hospital mortality during hospitalization compared to non-obese patients (HR = 2.65; 95% CI = 1.03 to 6.80; *p* = 0.042) (Figure 3).

### Interaction Between Obesity and Covariates

**FIGURE 4:**
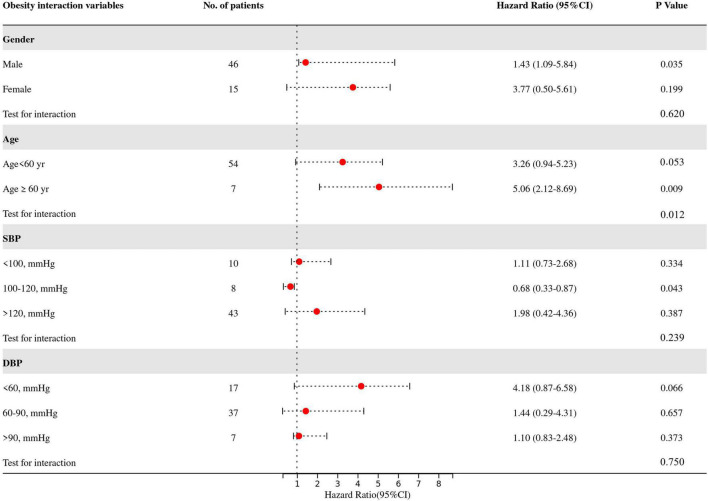
Multivariate-adjusted hazard ratios for death in patients in the obese group as compared with the non-obese group, according to four prognostic factors. Adjust variables: gender, age, SBP, DBP, heart rate, pre-operative delirium, LEVF 20–40%, time of ventricular fibrillation during surgery, aortic valve replacement, CABG, post-operative PLR, post-operative NLR, renal replacement therapy, and stroke. SBP, systolic blood pressure; DBP, diastolic blood pressure; LEVF, left ventricular ejection fraction; CABG, coronary artery bypass graft; PLR, platelet lymphocyte ratio; NLR, neutrocyte lymphocyte ratio.

The predetermined covariates were gender (men vs. women), age (<60 years vs. ≥ 60 years), SBP (<100 vs. 100–120 vs. > 120), and DBP (<60 vs. 60–90 vs. > 90) according to clinical guidelines and previous studies (17.25). We evaluated interactions between the four prognostic factors (gender, age, SBP, and DBP) (Figure 4) and obesity with a stepwise procedure for multivariate analysis. Figure 4 shows that there are significant interactions between age and obesity on in-hospital mortality (test for interaction, *p* = 0.012). Supplementary Tables 1, 2 present the subgroup analysis by age to analyze the effects of obesity and its effect on in-hospital mortality at different ages. We found that obesity was associated with an increased risk of in-hospital mortality in elderly patients (adjusted HR 5.06, 95% CI 2.12–8.69), while no obesity in younger patients protected them (adjusted HR 0.83, 95% CI 0.61–0.95).

## Discussion

This study results, comparing the role of obesity in open surgical repair of patients with AAD, are summarized as follows: (1) patients with obesity were younger and more prone to unstable blood pressure (SBP and DBP), pre-operative hypoxemia and delirium, prolonged operative time, and surgical wound deep infection; (2) in the fully adjusted model, we observed an increased risk of in-hospital mortality in patients with obesity after fine-tuning other covariates including age and sex; and (3) the interaction suggested that obesity was more likely to cause death in elderly patients (age ≥ 60), although it was more common in younger patients.

The prevalence of obesity is gradually getting younger as may be expected (2). We found that obesity was associated with a higher prevalence of hypertension and instability of blood pressure (extremely high or low), which may be mostly explained by the consequence of compromised aortic physiologic microregulation (25, 26). These clinical features may exacerbate aortic intima tear or rupture and may also reveal cardiac tamponade that inhibits cardiac pumping, which may partly account for poorer outcomes in patients with obesity. Hypoxemia, delirium, and wound infection may be associated with obesity-induced decreased lung compliance, obstructive sleep apnea, cervical or cerebral atherosclerosis, and fat liquefaction (27–29), while the long-term surgical procedure may increase the risk factors for death, such as inflammation, thrombosis, infection, etc. (30). We noticed that the obese group had longer operative time, CPB time, and hypothermic circulatory arrest time, which was consistent with previous reports (8, 31). On the one hand, the anatomical variation of obesity is more likely to increase the difficulty of intraoperative operations, such as anesthesia, thoracotomy, hemostasis, and suturing (32). On the other hand, the physiological abnormalities of obesity can easily break the intraoperative homeostasis of patients, such as unstable blood pressure, obesity-hypoventilation syndrome, and microcirculatory perfusion disorders (33, 34). All of these factors may lead to poorer surgical outcomes in patients with obesity. Consequently, surgeons need to pay more attention to the patient’s respiratory or airway status, mental state, inflammation, and change of dressing on the wound compared to non-obese patients. In this study, we also observed that patients with obesity had higher pre-operative RDW and NLR, which are risk factors for cardiovascular mortality described in previous studies (35–37). Their elevation may be associated with obesity-related lower-grade chronic inflammation and inflammatory mechanisms of aortic dissection (38–40). Of note, despite the differences in baseline characteristics being unavoidable, gender, past medical history besides hypertension, surgical details, and treatment management did not differ between the two groups.

The role of obesity in aortic disease has been controversial. Several previous studies have shown that obesity was not significantly associated with mortality through aortic dissection but only increases perioperative complications and ventilation time (7, 41, 42). The BMI of patients also had no effect on the early major adverse outcomes and mid-term survival by continuous grouping (8, 9). Whereas, BMI has also been reported to be a risk factor affecting the hospital mortality rate of aortic dissection undergoing Sun’s operation (43). Other studies suggested that morbid obesity significantly increased the mortality in open abdominal aortic surgery (41, 44, 45), which prompted the controversy still unresolved. On the one hand, BMI in patients with aortic dissection may present a non-linear relationship with adverse outcomes, which may be irregular and different from the U-shaped curve of the “obesity paradox” in cardiac surgery (3). This seems to explain why the effect of BMI on major adverse outcomes from aortic dissection varies among reports. On the other hand, these studies may have omitted the difference in obesity in the Chinese population. As mentioned earlier, the unified international BMI classification is not applicable to the Chinese population due to differences in ethnicity and living habits, which confound the association between BMI and AAD mortality. The previous reports may also have tended to associate obesity with better outcomes, such as hemiarch replacement and non-emergency surgery. In fact, due to anatomical lesions and high mortality rate, the current surgical procedure for patients with AAD is still emergency total arch replacement in order to prevent dissection extending, replace damaged aorta, and restore blood supply to vital organs as soon as possible (46).

The characteristics of such patients were reflected in our study. Although multiple risk factors for in-hospital mortality in patients with AAD were identified by LASSO regression, we still observed that obesity increased in-hospital mortality after full adjustment for confounders. When the study sample is less than 10 times the number of variables, the application of LASSO regression can mainly avoid overfitting and multicollinearity by using the tuning parameter (λ) for dimensionality reduction. The strong predictors screened by LASSO regression were substituted into the Cox proportional-hazard model to assess risks, which may also be closer to clinical practice (47–49). We found obesity to be a risk factor for in-hospital mortality in patients with AAD undergoing open surgical repair (HR = 2.65; 95% CI = 1.03 to 6.80; *p* = 0.042) and also unexpectedly found a significant interaction between obesity and age in in-hospital mortality effect (test for interaction, *p* = 0.012). Our results, if confirmed, suggest that elderly patients with AAD may be more susceptible to poor prognosis due to obesity, which may be omitted in previous studies leading to an unclear role of obesity. The elderly, with multiple comorbidities such as hypertension, diabetes, and arteriosclerosis, are prone to poor basic organ function and are inherently at higher risk of surgery. On this basis, the presence of obesity is likely to accelerate the deterioration of elderly patients due to complications. Of course, further design and verification will be necessary.

Obesity is a multifactorial disease that results from interactions between genetics and lifestyle. The heritability for obesity is known to be around 40%, while the remainder can be explained by lifestyle factors, which suggests that obesity is a modifiable risk factor (26, 50). Healthy living and weight management recommended by WHO are necessary for patients, because obesity may increase mortality at admission compared with the patient without obesity, despite unifying the surgical procedures. Compared to surgical options, the degree of patient obesity may also be a focus for surgeons, as obesity may upset the balance from onset to post-operative management, especially in elderly patients with obesity. In these patients, the surgeon may need to pay more attention to blood pressure stability, respiratory or airway status, mental status, inflammation, and change of dressing on the wound (51).

The study still has some limitations. First, as mentioned in the Methods, our study was based on the Chinese population, reducing the generalizability of the findings, and it is unclear whether it is applicable to other ethnicities. Second, survivorship bias may be unavoidable due to the high mortality of AAD. Third, our findings are derived from single-center observational data, and further multi-center studies and a high-quality meta-analysis should be carried out to provide more evidence. Simultaneously, limited by the sample size, we were unable to assess the interaction of stratified root procedure and concomitant surgery with multivariate adjustment, which will be addressed in our future studies. Finally, this study did not explore the effect of obesity degree and BMI on in-hospital mortality, as the Chinese criteria do not subdivide the obesity degree. The continuous grouping of BMI, linear or non-linear relationships, and optimal cutoff values for prediction were also not further explored, which may be addressed in our future studies.

## Conclusion

Obesity, interacting with age, increases the risk of in-hospital mortality in patients with AAD undergoing open surgical repair. Although more verification is needed, we believe that these findings provide further evidence for the treatment of AAD.

## Data Availability Statement

The original contributions presented in this study are included in the article/Supplementary Material, further inquiries can be directed to the corresponding author.

## Ethics Statement

The studies involving human participants were reviewed and approved by the Hospital Institutional Review Board of the Second Xiangya Hospital. Written informed consent for participation was not required for this study in accordance with the national legislation and the institutional requirements.

## Author Contributions

XP and XC drafted, revised, and reviewed the manuscript. XP, ZX, and GY conducted the statistical analysis and reviewed and revised the manuscript. ND and YZ organized the database. All authors significantly contributed to the conception, study design, execution, data acquisition, analysis, interpretation, approved the final version, and agreed on the journal and are responsible for this study.

## Conflict of Interest

The authors declare that the research was conducted in the absence of any commercial or financial relationships that could be construed as a potential conflict of interest.

## Publisher’s Note

All claims expressed in this article are solely those of the authors and do not necessarily represent those of their affiliated organizations, or those of the publisher, the editors and the reviewers. Any product that may be evaluated in this article, or claim that may be made by its manufacturer, is not guaranteed or endorsed by the publisher.

## Footnotes

1
http://www.empowerstats.com

2
http://www.r-project.org

## References

[1] He Y Lam T Jiang B Li L Sun D Wu L Changes in BMI before and during economic development and subsequent risk of cardiovascular disease and total mortality: a 35-year follow-up study in China. *Diabetes care.* (2014) 37:2540–7. 10.2337/dc14-0243 24947786

[2] Afshin A Forouzanfar M Reitsma M Sur P Estep K Lee A Health effects of overweight and obesity in 195 countries over 25 years. *N Engl J Med.* (2017) 377:13–27. 10.1056/NEJMoa1614362 28604169 PMC5477817

[3] Mariscalco G Wozniak M Dawson A Serraino G Porter R Nath M Body mass index and mortality among adults undergoing cardiac surgery: a nationwide study with a systematic review and meta-analysis. *Circulation.* (2017) 135:850–63. 10.1161/circulationaha.116.022840 28034901

[4] Steel N Ford J Newton J Davis A Vos T Naghavi M Changes in health in the countries of the UK and 150 english local authority areas 1990-2016: a systematic analysis for the global burden of disease study 2016. *Lancet.* (2018) 392:1647–61. 10.1016/s0140-6736(18)32207-4 30497795 PMC6215773

[5] Wang J Yu W Zhai G Liu N Sun L Zhu J. Independent risk factors for postoperative AKI and the impact of the AKI on 30-day postoperative outcomes in patients with type A acute aortic dissection: an updated meta-analysis and meta-regression. *J Thorac Dis.* (2018) 10:2590–8. 10.21037/jtd.2018.05.47 29997920 PMC6006120

[6] Zhou J Pan J Yu Y Huang W Lai Y Liang W Independent risk factors of hypoxemia in patients after surgery with acute type A aortic dissection. *Ann Palliat Med.* (2021) 10:7388–97. 10.21037/apm-21-1428 34263634

[7] Pan X Lu J Cheng W Yang Y Zhu J Jin M. Independent factors related to preoperative acute lung injury in 130 adults undergoing Stanford type-A acute aortic dissection surgery: a single-center cross-sectional clinical study. *J Thorac Dis.* (2018) 10:4413–23. 10.21037/jtd.2018.06.140 30174890 PMC6105969

[8] Li Y Jiang H Xu H Li N Zhang Y Wang G Impact of a higher body mass index on prolonged intubation in patients undergoing surgery for acute thoracic aortic dissection. *Heart Lung Circ.* (2020) 29:1725–32. 10.1016/j.hlc.2020.02.008 32224088

[9] Kreibich M Rylski B Bavaria J Branchetti E Dohle D Moeller P Outcome after operation for aortic dissection type a in morbidly obese patients. *Ann Thorac Surg.* (2018) 106:491–7. 10.1016/j.athoracsur.2018.03.035 29673638

[10] Rogge B Cramariuc D Lønnebakken M Gohlke-Bärwolf C Chambers J Boman K Effect of overweight and obesity on cardiovascular events in asymptomatic aortic stenosis: a SEAS substudy (Simvastatin Ezetimibe in Aortic Stenosis). *J Am Coll Cardiol.* (2013) 62:1683–90. 10.1016/j.jacc.2013.04.081 23770175

[11] McGorrian C Yusuf S Islam S Jung H Rangarajan S Avezum A Estimating modifiable coronary heart disease risk in multiple regions of the world: the INTERHEART Modifiable Risk Score. *Eur Heart J.* (2011) 32:581–9. 10.1093/eurheartj/ehq448 21177699

[12] Bossone E LaBounty TM Eagle KA. Acute aortic syndromes: diagnosis and management, an update. *Eur Heart J.* (2018) 39:739–49d. 10.1093/eurheartj/ehx319 29106452

[13] Sun L Qi R Zhu J Liu Y Zheng J. Total arch replacement combined with stented elephant trunk implantation: a new “standard” therapy for type a dissection involving repair of the aortic arch? *Circulation.* (2011) 123:971–8. 10.1161/circulationaha.110.015081 21339481

[14] Gu D Reynolds K Wu X Chen J Duan X Reynolds R Prevalence of the metabolic syndrome and overweight among adults in China. *Lancet.* (2005) 365:1398–405. 10.1016/s0140-6736(05)66375-1 15836888

[15] Chen X Ding G Xu L Li P. A glimpse at the metabolic research in China. *Cell Metabol.* (2021) 33:2122–5. 10.1016/j.cmet.2021.09.014 34619075

[16] Lightwood J Bibbins-Domingo K Coxson P Wang Y Williams L Goldman L. Forecasting the future economic burden of current adolescent overweight: an estimate of the coronary heart disease policy model. *Am J Public Health.* (2009) 99:2230–7. 10.2105/ajph.2008.152595 19833999 PMC2775763

[17] Erbel R Aboyans V Boileau C Bossone E Bartolomeo R Eggebrecht H 2014 ESC Guidelines on the diagnosis and treatment of aortic diseases: Document covering acute and chronic aortic diseases of the thoracic and abdominal aorta of the adult. The Task Force for the Diagnosis and Treatment of Aortic Diseases of the European Society of Cardiology (ESC). *Eur Heart J.* (2014) 35:2873–926. 10.1093/eurheartj/ehu281 25173340

[18] Sun L Qi R Chang Q Zhu J Liu Y Yu C Surgery for acute type A dissection using total arch replacement combined with stented elephant trunk implantation: experience with 107 patients. *J Thorac Cardiovasc Surg.* (2009) 138:1358–62. 10.1016/j.jtcvs.2009.04.017 19660407

[19] Ma W Zhu J Zheng J Liu Y Ziganshin B Elefteriades J Sun’s procedure for complex aortic arch repair: total arch replacement using a tetrafurcate graft with stented elephant trunk implantation. *Ann Cardiothorac Surg.* (2013) 2:642–8. 10.3978/j.issn.2225-319X.2013.09.03 24109575 PMC3791186

[20] Tan L Xiao J Zhou X Shen K Li F Luo J Untreated distal intimal tears may be associated with paraplegia after total arch replacement and frozen elephant trunk treatment of acute Stanford type A aortic dissection. *J Thorac Cardiovasc Surg.* (2019) 158: 343-50.e1. 10.1016/j.jtcvs.2018.08.111 30396731

[21] Blazek K van Zwieten A Saglimbene V Teixeira-Pinto A. A practical guide to multiple imputation of missing data in nephrology. *Kidney Int.* (2021) 99:68–74. 10.1016/j.kint.2020.07.035 32822702

[22] Bernhardt P. Model validation and influence diagnostics for regression models with missing covariates. *Stat Med.* (2018) 37:1325–42. 10.1002/sim.7584 29318652

[23] Yang G Peng W Zhou Y He H Pan X Li X Admission systolic blood pressure and in-hospital mortality in acute type a aortic dissection: a retrospective observational study. *Front Med.* (2021) 8:542212. 10.3389/fmed.2021.542212 34354998 PMC8329236

[24] Liang W Liang H Ou L Chen B Chen A Li C Development and validation of a clinical risk score to predict the occurrence of critical illness in hospitalized patients with COVID-19. *JAMA Intern Med.* (2020) 180:1081–9. 10.1001/jamainternmed.2020.2033 32396163 PMC7218676

[25] Nernpermpisooth N Qiu S Mintz J Suvitayavat W Thirawarapan S Rudic D Obesity alters the peripheral circadian clock in the aorta and microcirculation. *Microcirculation.* (2015) 22:257–66. 10.1111/micc.12192 25660131 PMC4532430

[26] Kim M Kim W Khera A Kim J Yon D Lee S Association between adiposity and cardiovascular outcomes: an umbrella review and meta-analysis of observational and Mendelian randomization studies. *Eur Heart J.* (2021) 42:3388–403. 10.1093/eurheartj/ehab454 34333589 PMC8423481

[27] De Santis Santiago R Teggia Droghi M Fumagalli J Marrazzo F Florio G Grassi L High Pleural Pressure Prevents Alveolar Overdistension and Hemodynamic Collapse in ARDS with Class III Obesity. *Am J Respir Critic Care Med.* (2020) 203:575–584. 10.1164/rccm.201909-1687OC 32876469 PMC7924574

[28] Le Guezennec X Brichkina A Huang Y Kostromina E Han W Bulavin D. Wip1-dependent regulation of autophagy, obesity, and atherosclerosis. *Cell Metabol.* (2012) 16:68–80. 10.1016/j.cmet.2012.06.003 22768840

[29] Thelwall S Harrington P Sheridan E Lamagni T. Impact of obesity on the risk of wound infection following surgery: results from a nationwide prospective multicentre cohort study in England. *Clin Microbiol Infect.* (2015) 21:.e1001–8. 10.1016/j.cmi.2015.07.003 26197212

[30] Nepogodiev D Chapman S Glasbey J Kelly M Khatri C Drake T Determining Surgical Complications in the Overweight (DISCOVER): a multicentre observational cohort study to evaluate the role of obesity as a risk factor for postoperative complications in general surgery. *BMJ Open.* (2015) 5:e008811. 10.1136/bmjopen-2015-008811 26195471 PMC4513439

[31] Nagendran J Moore M Norris C Khani-Hanjani A Graham M Freed D The varying effects of obesity and morbid obesity on outcomes following cardiac transplantation. *Int J Obes.* (2016) 40:721–4. 10.1038/ijo.2016.20 26853917

[32] Poirier P Alpert M Fleisher L Thompson P Sugerman H Burke L Cardiovascular evaluation and management of severely obese patients undergoing surgery: a science advisory from the American Heart Association. *Circulation.* (2009) 120:86–95. 10.1161/circulationaha.109.192575 19528335

[33] den Os MM van den Brom CE van Leeuwen ALI Dekker NAM. Microcirculatory perfusion disturbances following cardiopulmonary bypass: a systematic review. *Critic Care.* (2020) 24:218. 10.1186/s13054-020-02948-w 32404120 PMC7222340

[34] Samad F Ruf W. Inflammation, obesity, and thrombosis. *Blood.* (2013) 122:3415–22. 10.1182/blood-2013-05-427708 24092932 PMC3829115

[35] Bedel C Selvi F. Association of Platelet to Lymphocyte and Neutrophil to Lymphocyte Ratios with In-Hospital Mortality in Patients with Type A Acute Aortic Dissection. *Braz J Cardiovasc Surg.* (2019) 34:694–8. 10.21470/1678-9741-2018-0343 31545575 PMC6894039

[36] Baggen V van den Bosch A van Kimmenade R Eindhoven J Witsenburg M Cuypers J Red cell distribution width in adults with congenital heart disease: a worldwide available and low-cost predictor of cardiovascular events. *Int J Cardiol.* (2018) 260:60–5. 10.1016/j.ijcard.2018.02.118 29525069

[37] Bhutta H Agha R Wong J Tang T Wilson Y Walsh S. Neutrophil-lymphocyte ratio predicts medium-term survival following elective major vascular surgery: a cross-sectional study. *Vasc Endovasc Surg.* (2011) 45:227–31. 10.1177/1538574410396590 21289130

[38] Elisia I Lam V Cho B Hay M Krystal G. Exploratory examination of inflammation state, immune response and blood cell composition in a human obese cohort to identify potential markers predicting cancer risk. *PLoS One.* (2020) 15:e0228633. 10.1371/journal.pone.0228633 32027700 PMC7004330

[39] Zhou L Lin S Zhang F Ma Y Fu Z Gong Y The Correlation Between RDW, MPV and Weight Indices After Metabolic Surgery in Patients with Obesity and DM/IGR: follow-Up Observation at 12 Months. *Diabetes Ther.* (2020) 11:2269–81. 10.1007/s13300-020-00897-9 32789779 PMC7509025

[40] Son B Sawaki D Tomida S Fujita D Aizawa K Aoki H Granulocyte macrophage colony-stimulating factor is required for aortic dissection/intramural haematoma. *Nat Commun.* (2015) 6:6994. 10.1038/ncomms7994 25923510

[41] Giles K Wyers M Pomposelli F Hamdan A Ching Y Schermerhorn M. The impact of body mass index on perioperative outcomes of open and endovascular abdominal aortic aneurysm repair from the National Surgical Quality Improvement Program, 2005-2007. *J Vasc Surg.* (2010) 52:1471–7. 10.1016/j.jvs.2010.07.013 20843627 PMC3005989

[42] Chen X Zhou J Fang M Yang J Wang X Wang S Incidence-and In-hospital Mortality-Related Risk Factors of Acute Kidney Injury Requiring Continuous Renal Replacement Therapy in Patients Undergoing Surgery for Acute Type a Aortic Dissection. *Front Cardiovasc Med.* (2021) 8:749592. 10.3389/fcvm.2021.749592 34888362 PMC8650701

[43] Zhang Y Chen T Chen Q Min H Nan J Guo Z. Development and evaluation of an early death risk prediction model after acute type A aortic dissection. *Ann Translat Med.* (2021) 9:1442. 10.21037/atm-21-4063 34733994 PMC8506734

[44] Khorgami Z Sclabas G Aminian A Lau P Chow G Malgor R Mortality in open abdominal aortic surgery in patients with morbid obesity. *Surg Obes Relat Dis.* (2019) 15:958–63. 10.1016/j.soard.2019.03.044 31097382

[45] Radak D Tanaskovic S Neskovic M. The Obesity-associated Risk in Open and Endovascular Repair of Abdominal Aortic Aneurysm. *Curr Pharmaceutic Design.* (2019) 25:2033–7. 10.2174/1381612825666190710112844 31291872

[46] Bossone E Eagle K. Epidemiology and management of aortic disease: aortic aneurysms and acute aortic syndromes. *Nat Rev Cardiol.* (2021) 18:331–48. 10.1038/s41569-020-00472-6 33353985

[47] Frank L Heiser W. Feature selection in feature network models: finding predictive subsets of features with the Positive Lasso. *Br J Mathematic Stat psychol.* (2008) 61:1–27. 10.1348/000711006x119365 18482473

[48] Friedman J Hastie T Tibshirani R. Regularization Paths for Generalized Linear Models via Coordinate Descent. *J Stat Softw.* (2010) 33:1–22. 20808728 PMC2929880

[49] Lee T Chao P Ting H Chang L Huang Y Wu J Using multivariate regression model with least absolute shrinkage and selection operator (LASSO) to predict the incidence of Xerostomia after intensity-modulated radiotherapy for head and neck cancer. *PLoS One.* (2014) 9:e89700. 10.1371/journal.pone.0089700 24586971 PMC3938504

[50] Locke A Kahali B Berndt S Justice A Pers T Day F Genetic studies of body mass index yield new insights for obesity biology. *Nature.* (2015) 518:197–206. 10.1038/nature14177 25673413 PMC4382211

[51] Nienaber C Clough R Sakalihasan N Suzuki T Gibbs R Mussa F Aortic dissection. *Nat Rev Dis Prim.* (2016) 2:16071. 10.1038/nrdp.2016.71 27560366

