# Supplementary material for: Obesity Increases In-Hospital Mortality of Acute Type A Aortic Dissection Patients Undergoing Open Surgical Repair: A Retrospective Study in the Chinese Population
Source: Front Cardiovasc Med. 2022 Jul 12;9:899050. doi: 10.3389/fcvm.2022.899050 (PMC9315262; doi:10.3389/fcvm.2022.899050)
Supplement: Supplementary file 1 [file Data_Sheet_1.docx]

**Supplementary material**

Table S1. The important characteristics of patients according to age subgroup.

| Variables | Age <60 (n=251) | Age ≥60 (n=38) | P-value |
| --- | --- | --- | --- |
| **Gender** |  |  | 0.004 |
| Male | 194 (77.29%) | 21 (55.26%) |  |
| Female | 57 (22.71%) | 17 (44.74%) |  |
| BMI, kg/m^2^ | 24.94 ± 3.10 | 24.19 ± 3.35 | 0.167 |
| Obese | 54 (21.51%) | 7 (18.42%) | 0.663 |
| SBP,mmHg | 133.51 ± 27.89 | 131.74 ± 29.57 | 0.718 |
| < 100.00 | 24 (9.56%) | 6 (15.79%) | 0.241 |
| 100.00 - 120.00 | 65 (25.90%) | 9 (23.68%) | 0.771 |
| > 120.00 | 162 (64.54%) | 23 (60.53%) | 0.631 |
| DBP,mmHg | 75.59 ± 15.41 | 72.45 ± 12.90 | 0.233 |
| < 60.00 | 33 (13.15%) | 7 (18.42%) | 0.380 |
| 60.00 - 90.00 | 175 (69.72%) | 29 (76.32%) | 0.406 |
| > 90.00 | 43 (17.13%) | 2 (5.26%) | 0.060 |
| Heart rate / min | 81.74 ± 15.02 | 74.82 ± 13.03 | 0.008 |
| Delirium | 5 (1.99%) | 3 (7.89%) | 0.039 |
| LVEF 20.00 - 40.00 | 2 (0.80%) | 0 (0.00%) | 0.998 |
| **Root procedure** |  |  |  |
| Bentall | 221(88.05%) | 34(89.47%) | 0.897 |
| David | 23(9.16%) | 3(7.89%) | 0.651 |
| Wheat | 7(2.79%) | 1(2.63%) | 0.980 |
| **Aortic arch procedure** |  |  |  |
| Total arch replacement | 242 (96.41%) | 28 (73.68%) | <0.001 |
| Hemi-arch replacement | 5 (1.99%) | 4 (10.53%) | 0.005 |
| **Concomitant procedure** |  |  |  |
| Aortic valve replacement | 6 (2.39%) | 1 (2.63%) | 0.928 |
| CABG | 22 (8.76%) | 8 (21.05%) | 0.021 |
| Ventricular fibrillation,seconds | 34.58 ± 21.78 | 27.37 ± 21.51 | 0.058 |
| sPLR | 155.78 ± 85.33 | 139.54 ± 67.49 | 0.283 |
| sNLR | 11.79 ± 4.33 | 11.71 ± 4.32 | 0.920 |
| **Complications** |  |  |  |
| Respiratory infection | 33 (13.25%) | 3 (9.09%) | 0.501 |
| Surgical wound deep infection | 9 (3.60%) | 1 (3.03%) | 0.868 |
| Renal replacement therapy | 47 (18.88%) | 6 (18.18%) | 0.924 |
| Paraplegia | 8 (3.21%) | 0 (0.00%) | 0.602 |
| Temporary neurological dysfunction | 9 (3.61%) | 3 (9.09%) | 0.143 |
| Stroke | 10 (4.02%) | 3 (8.57%) | 0.227 |
| Hospital stay,day | 20.27 ± 9.42 | 17.89 ± 10.79 | 0.156 |
| ICU stay rate | 0.42 ± 0.19 | 0.43 ± 0.25 | 0.647 |
| In-hospital mortality | 27 (10.76%) | 12 (31.58%) | <0.001 |

Data are presented as n (%) or mean (IQR). **Abbreviations:** BMI, body mass index; SBP, systolic blood pressure; DBP, diastolic blood pressure; LVEF, left ventricular ejection fraction; CABG, coronary artery bypass graft; sPLR, postoperative platelet lymphocyte ratio; sNLR, postoperative neutrocyte lymphocyte ratio; ICU, intensive care unit.

Table S2. The effects of obesity on in-hospital mortality at different ages.

| Interaction variables | No. Of patients | Univariate analysis | | Multivariate analysis | |
| --- | --- | --- | --- | --- | --- |
|  |  | Unadjusted HR (95% CI) | P | Adjusted HR (95% CI) | P |
| **Age <60** |  |  |  |  |  |
| Non-obese | 197 | 0.78 (0.54, 0.89) | 0.033 | 0.83 (0.61, 0.95) | 0.028 |
| Obese | 54 | 1.93 (0.83, 4.45) | 0.125 | 3.01 (0.88, 5.26) | 0.078 |
| **Age ≥60** |  |  |  |  |  |
| Non-obese | 31 | 1.80 (0.70, 4.66) | 0.223 | 2.25 (0.67, 7.57) | 0.188 |
| Obese | 7 | 3.88 (2.06, 8.10) | <0.001 | 5.06 (2.12, 8.69) | 0.009 |

Adjust variables: gender, SBP, DBP, heart rate, preoperative delirium, LEVF 20%-40%, time of ventricular fibrillation during surgery, aortic valve replacement, CABG, postoperative PLR, postoperative NLR, renal replacement therapy, stroke. **Abbreviations:** SBP, systolic blood pressure; DBP, diastolic blood pressure; LEVF, left ventricular ejection fraction; CABG, coronary artery bypass graft; PLR, platelet lymphocyte ratio; NLR, neutrocyte lymphocyte ratio.


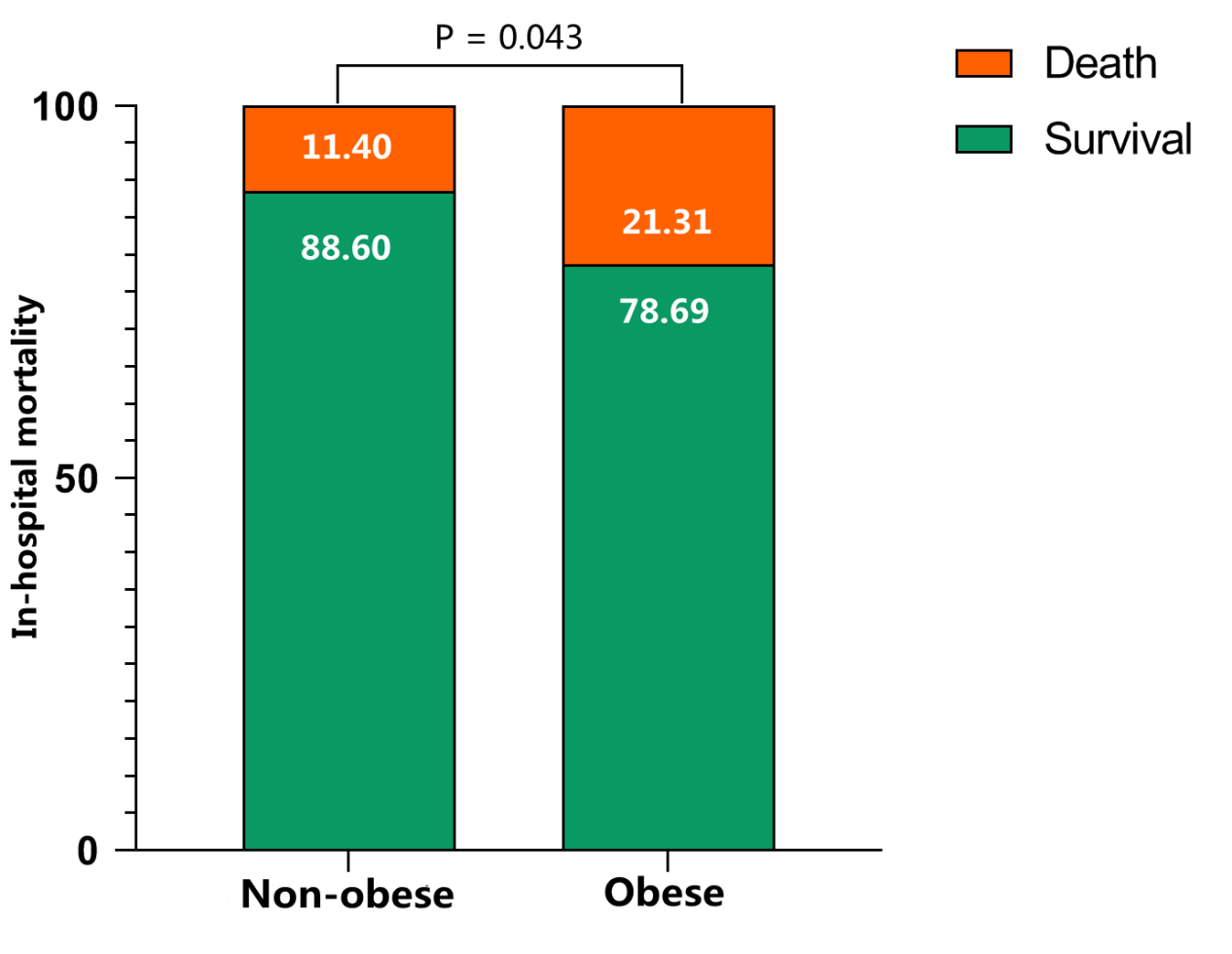


**Figure S1**. Comparison of in-hospital mortality stratified by obesity.


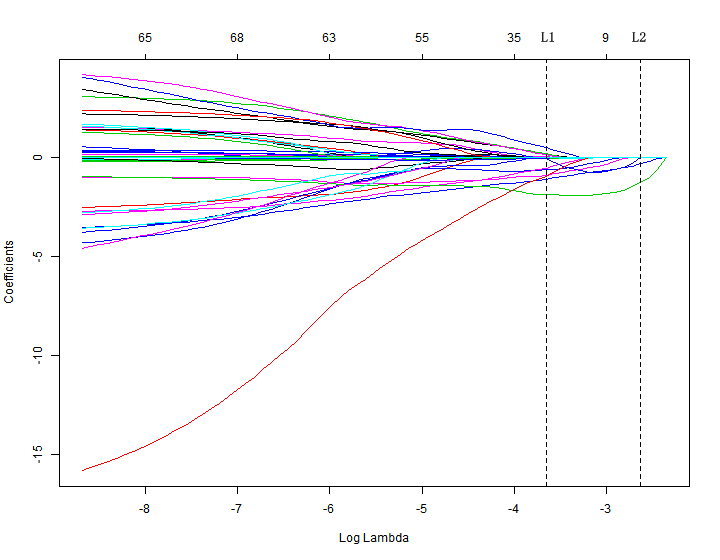

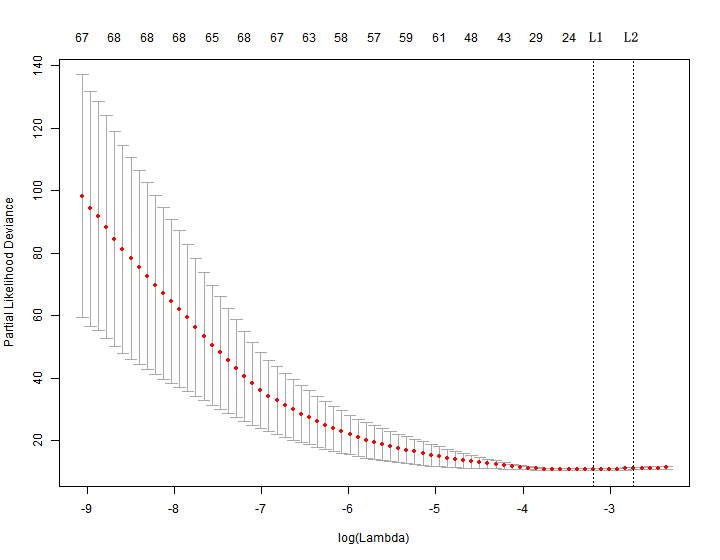


(A) (B)

**Figure S2**. Feature selection using the least absolute shrinkage and selection operator (LASSO) binary logistic regression model. (A) LASSO uses the L1 penalty to select stronger predictors of the outcome while shrinking the coefficient for weaker predictors towards zero. (B) Tuning parameter (λ) selection in the LASSO model used 10-fold cross-validation via minimum criteria. (Folds for cross-validation were sampled on the person-level and stratified by event status)
